# *Salmonella enterica* Serovar Enteritidis Liver Abscess after Blunt Abdominal Trauma

**DOI:** 10.4274/balkanmedj.2016.1199

**Published:** 2017-09-29

**Authors:** Agata Ladic, Igor Petrovic, Ante Gojevic, Emil Kinda, Ivo Coza

**Affiliations:** 1 Division of Gastroenterology and Hepatology, Zagreb University Hospital Centre, Zagreb, Croatia; 2 Division of Abdominal Surgery, Zagreb University Hospital Centre, Zagreb, Croatia; 3 Department Abdominal Surgery, Zadar General Hospital, Zadar, Croatia

**Keywords:** Salmonella enteritidis, liver, abscess

## Abstract

**Background:**

*Salmonella enterica* serovar Enteritidis is among the most reported serotypes of *Salmonella* species worldwide, but is rarely reported as the causative agent of a liver abscess.

**Case Report:**

We present a patient with an abdominal blunt trauma. An initial abdominal computed tomography scan revealed a rupture of the right kidney and of the liver. Two days later, his physical state deteriorated and a new computed tomography scan was obtained. An extremely large 8-centimetre liver abscess was verified. Due to the unsatisfying response to antibiotic therapy and percutaneous drainage, we operated on the patient. An appendectomy, cholecystectomy and bisegmental liver resection were performed. An intraoperative swab from the abscess was positive for *Salmonella* enterica serovar Enteritidis. The patient was given intravenous ciprofloxacine. The post-operative course was complicated by a Coagulase-negative *Staphylococcus* infection of the wound, which improved with antibiotic therapy.

**Conclusion:**

Blunt abdominal trauma may initiate an unpredictable course of the disease in chronic *Salmonella* carriers.

Blunt abdominal trauma is one of the leading causes of morbidity and mortality across all age groups, notably in people younger than 45, with the spleen and the liver being the most commonly injured abdominal organs (1). These blunt trauma complications include: haematoma rupture, intra-abdominal abscess, bowel obstruction or ileus, biliary leakage and/or biloma and abdominal compartment syndrome. If the abscess develops, the infected organism usually reflects a bowel flora, with the most frequent isolates being aerobic Gram-negative bacilli (such as *Escherichia coli* and *Klebsiella*) and anaerobes (*Bacteroides fragilis*) (2).

*Salmonella* is a rod-shaped bacterium that belongs to the *Enterobacteriacea* family. It usually affects the gastrointestinal tract, but occasionally crosses the intestinal mucosal barrier and localizes in other abdominal organs, bringing about a spectrum of clinical presentations: from mild gastroenteritis to bacteraemia and abdominal abscesses. The true rate of *Salmonella* bacteraemia is unknown due to an often mild or microbiologically non-confirmed infection (3). There is a lack of information on the *Salmonella* enterica serovar Enteritidis reservoir in chronic human carriers, but as with *S. enterica* serovar Typhi, which persists primarily in the gall bladder, it is quite likely that serovar Enteritidis inhabits the gall bladder as well (3).

Salmonellosis has decreased during the last 10 years in western Europe and in the US, while in Croatia it is still one of the leading causes of bacterial food poisoning (4).

Current recommendations for treating a Salmonella abscess reflect the treatment for all pyogenic abscesses: percutaneous or surgical drainage in conjunction with antibiotic therapy, which should be started as soon as the diagnosis is suspected.

We present a rare case of a liver abscess following abdominal blunt trauma - to the best of our knowledge, there has only been one case of a *S. enterica* serovar Enteritidis liver abscess following blunt trauma reported so far (5).

## CASE PRESENTATION

A 27-year-old patient, who lived in the Adriatic coastal area, was admitted to the regional hospital after being injured in a motorbike accident, in which he acquired a blunt abdominal trauma to the right hemiabdomen, a clavicular fracture, serial rib fractures and fractures of the metacarpal bones. His medical history was unremarkable. He did not have any chronic viral infections, and the serology for HIV was negative.

**FIG. 1.**
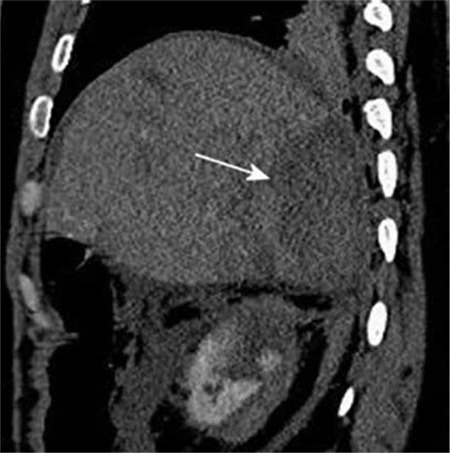
Contrast-enhanced computed tomography sagittal scan reconstruction showing subcapsular haematoma in the right liver lobe (arrow).

Upon admission, the patient was haemodynamically stable. The initial laboratory results revealed anaemia (haemoglobin 98 g/L), hepatic injury (aspartate transaminase 968 U/L, alanine transaminase 1520 U/L) and kidney injury (creatinine 147 µmol/L). The computed tomography (CT) scan revealed a right kidney and right liver lobe rupture, with a subcapsular liver haematoma (Figure 1).

Two days later his physical state deteriorated, with dyspnoea, tachycardia and hypotension. Laboratory tests represented a sepsis: a leukocyte count of 5.200/mm^3^ with 30% of non-segmented polymorphonuclear leukocytes, haemoglobin 89 g/L, platelet count 123/mm^3^, C-reactive protein 184 mg/L, aspartate transaminase 1165 U/L, alanine transaminase 1809 U/L, creatinine 155 µmol/L, serum procalcitonine 26.24 ng/mL and lactate 4.1 mmol/L. There were no other serum abnormalities. Intravenous meropenem 3x1 g (Meronem, AstraZeneca Limited; Cheshire, United Kingdom) and vancomycin 2x1 g (Edicin, Lek farmacevtska druzba d.d.; Ljubljana, Slovenia) were administered. A control CT scan revealed gas inside the huge lesion of the right liver lobe, i.e. it revealed a newly formed liver abscess.

Because of the physical deterioration, the patient was sent to a tertiary-care clinic for further medical care. 

**FIG. 2.**
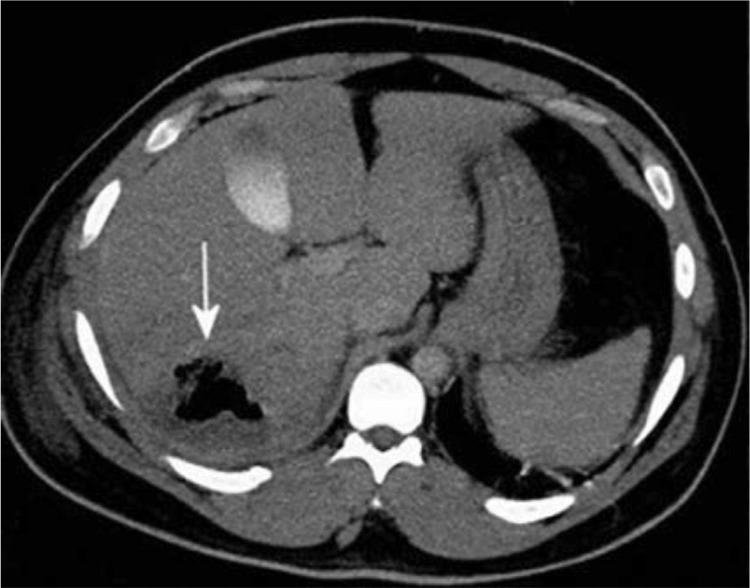
Contrast-enhanced computed tomography axial scan at follow-up showing a huge gas-forming abscess in the right liver lobe (arrow).

On admission to our clinic for surgery, the patient was haemodynamically stable with a noradrenaline (Arterenol, Sanofi-Aventis; Frankfurt/Main, Germany) support. On physical examination there was a diminished breath sound in the basal part of the right lung lobe. A contrast-enhanced CT scan of the thorax, abdomen and pelvic region was obtained. It confirmed a huge eight-centimetre abscess (Figure 2). Despite the antibiotic treatment, the patient was continuously febrile. C-reactive protein was repeatedly elevated, up to 299 mg/L (Table 1). Blood cultures revealed *S. enterica* serovar Enteritidis. The bacterium was isolated using Matrix-Assisted Laser Desorption Ionization Time-of-Flight Mass Spectrometry, while antimicrobial susceptibility testing was performed by disc diffusion according to the European Committee on Antimicrobial Sensitivity Testing breakpoints (6). The isolate was sensitive to amoxicillin, ceftriaxone, ciprofloxacine and trimethoprim/sulfamethoxazole, so the antibiotic therapy was switched to ciprofloxacine, 2x400 mg (Ciprinol, Krka d.d.; Novo Mesto, Slovenia) intravenously daily. Ultrasound-guided percutaneous abscess drainage was performed with only a small quantity of the material obtained.

In light of the constant elevation of inflammatory markers and not entirely successful percutaneous liver drainage, an operation was indicated. It began as a laparoscopic procedure, but following CO_2_ insufflation, the patient became tachycardic, and the operation was converted to midline laparotomy extended to the right subcostal area.

A liver bisegmentectomy of the sixth and seventh segment was performed, in addition to cholecystectomy and appendectomy. An intraoperative swab from the liver abscess was positive for *S. enterica* serovar Enteritidis.

The post-operative course was uneventful. The patient continued with intravenous ciprofloxacine treatment (Ciprinol, Krka d.d.; Novo Mesto, Slovenia), 2x400 mg daily. Two weeks later, the patient was febrile again, with a body temperature of 39 °C, with chills and cold sweats. After a thorough examination, we noticed a skin wound and took a swab - it was positive for a Coagulase-negative *Staphylococcus*, sensitive to vancomycin. The ciprofloxacine treatment was changed after 16 days to intravenous vancomycin 2x1 g (Edicin, Lek farmacevtska druzba d.d.; Ljubljana, Slovenia) and piperacillin/tazobactam 3x4.5 g (Tazocin, Wyeth Lederle S.p.A.; Catania, Italy) daily, according to the recommendation of the clinical pharmacologist. The rest of the post-operative course was uneventful and the patient was discharged home four weeks after the operation. Written informed consent was obtained from the patient.

## DISCUSSION

*Salmonella* infection is a very common bacterial infection, obtained through contaminated water or food. More than 2.500 *Salmonella* serovars exist; the serovar Enteritidis is among the most commonly reported serovars of human salmonellosis in industrialized countries (4). It causes diverse clinical syndromes - from unnoticed infection or gastroenteritis with febrile episodes in otherwise healthy subjects to complicated intra-abdominal infections in immune-compromised patients (3). Chronic carriage of non-typhoidal *Salmonella* serovars (NTS), such as serovar Enteritidis, occurs in 0.5% of cases, compared to 3% of typhoidal serovars (7). A growing number of clinical cases report on chronic NTS carriage in immunocompetent patients. According to the available data from our country (Croatia), the prevalence of a chronic *Salmonella* carrier state is 3% (8).

Both NTS and typhoid serovars initially adhere to the small intestine epithelium, where they encounter gastrointestinal microbiome - a huge body of intestinal bacteria that acts as a defence mechanism against multiple pathogens. In salmonellosis, it protects the gut by producing toxic metabolites that repress *Salmonella* virulence gene expression and mediate bacterial gut clearance (9). Recent reports have suggested, though, that certain NTS serovars, by inducing intestinal inflammation, develop the ability to consume non-fermentable substrates, in contrast to the host microbiota, which relies on fermentative metabolism. This gives *Salmonella* an advantage in terms of growth over the host microbiome (9). After the initial intestinal infection, typhoid serovars inhabit the gall bladder, where they live in a dormant-like state (3). There is no strong evidence that serovar Enteritidis inhabits the gall bladder as well, but it is known that it can be cultivated from bile and faeces. It can reach the gall bladder through blood drainage but also from the bowel along the bile ducts (3).

Hepatic abscesses are uncommon, according to a North American study that found an incidence of 2.3 cases per 100.000 inhabitants (10). Predisposing factors for the formation of abscesses are cholelythiasis, intrahepatic haematomas and hepatocellular carcinoma, as well as blunt and penetrating trauma to the abdomen. Hepatic abscesses are mostly treated conservatively - with percutaneous or surgical drainage, in conjunction with antibiotic therapy. The duration of antibiotic therapy depends on the underlying symptoms and possible complications and should be accompanied by careful imaging and monitoring for complications.

*S. enterica* serovar Enteritidis is rarely described as a liver abscess pathogen (2,5). In contrast to all other *Salmonella* serovars, *S. enterica* serovar Enteritidis has the potential to form a gas from carbohydrates. The gas accumulation results in an impaired transport of nutrients in adjacent tissues and promotes tissue destruction (2). This makes *S. enterica* serovar Enteritidis an extremely noxious pathogen. Cerwenka et al. (5) presented a case 20 years ago in which their patient had a blunt abdominal trauma, after which he developed a small biliary fistula that filled the abscess with *Salmonella*-infected biliary content. Our patient was young and previously healthy, but most likely a chronic *Salmonella* carrier, who probably went through the same pathogenic process - his abscess was filled with bacteria through injured small bile ducts.

Although the current recommendations are for conservative treatment of a liver abscess for as long as is possible, we decided on surgery very early on. This decision was based upon: a) extremely rapid deterioration with dysregulated host response and organ dysfunction, b) unsatisfying results of percutaneous liver drainage. The surgical approach was chosen due to the fact that the abscess was filled not only with pus, but also with necrotic and haemorrhagic content.

In conclusion, *Salmonella* may carry an unpredictable disease course, especially in immune-compromised patients. A *Salmonella* liver abscess should be considered in regions/countries with a higher incidence of chronic carriers. The most appropriate approach to this problem lies in the primary prevention of foodborne illnesses.

## Figures and Tables

**Table 1:**
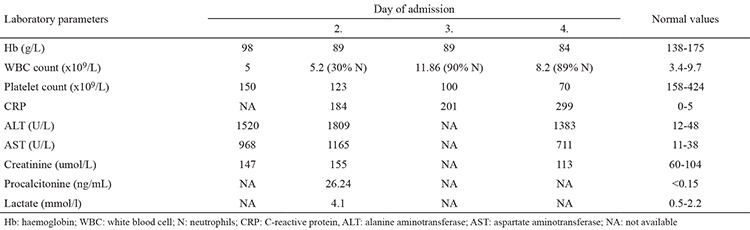
Preoperative laboratory parameters of the patient with post-traumatic liver abscess (S. enterica serovar Enteritidis)
